# Controlled Synthesis of Liquid-Crystalline Polymers Under Ambient Conditions by Red-Light-Driven ATRP

**DOI:** 10.1021/acs.macromol.5c03040

**Published:** 2026-02-16

**Authors:** Kaito Takahashi, Kaho Nakano, Xiaolei Hu, Khidong Kim, Hironobu Murata, Krzysztof Matyjaszewski, Atsushi Shishido, Shoichi Kubo

**Affiliations:** † Laboratory for Chemistry and Life Science, Institute of Integrated Research, 693022 Institute of Science Tokyo, Yokohama 226-8501, Japan; ‡ Department of Chemical Science and Engineering, School of Materials and Chemical Technology, Institute of Science Tokyo, Meguro, Tokyo 152-8552, Japan; § Department of Chemistry, 6612 Carnegie Mellon University, Pittsburgh, Pennsylvania 15213, United States; ∥ Research Center for Autonomous Systems Materialogy, Institute of Integrated Research, Institute of Science Tokyo, Yokohama 226-8501, Japan

## Abstract

Red-light-driven atom transfer radical polymerization (ATRP) using methylene blue as a photocatalyst has attracted considerable attention due to its high efficiency and oxygen tolerance. However, its application to functional polymers remains unexplored. Here we demonstrate the synthesis of liquid-crystalline polymers (LCPs) under ambient conditions using red-light-driven ATRP. Well-controlled polymerizations with high monomer conversions were achieved within several hours. The polymer growth could be precisely regulated by alternating the light irradiation on and off. Molecular weights were tuned by adjusting the polymerization conditions, affording LCPs with narrow molecular weight distributions. A clear relationship between molecular weight and LC behavior was observed. The method was extended to various mesogenic monomers and polymerizable groups, confirming its broad applicability. Moreover, controlled polymerization was achieved using accessible light sources such as sunlight and smartphone LED flashlights. This versatile approach enables efficient LCP synthesis and facilitates exploration of structure–property relationships in functional soft materials.

## Graphical abstract

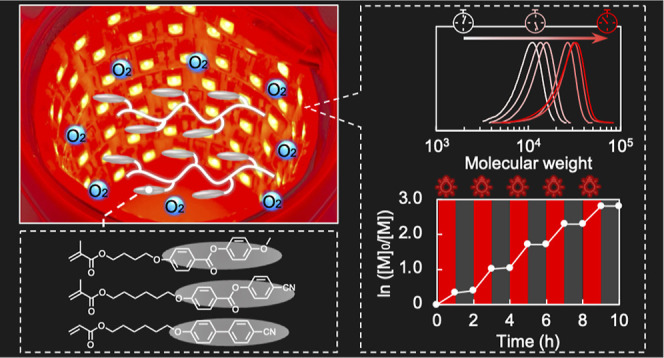

## Introduction

Reversible-deactivation radical polymerization (RDRP), including atom transfer radical polymerization (ATRP), reversible addition–fragmentation chain transfer polymerization (RAFT), and nitroxide-mediated polymerization (NMP), enables precise control over polymer chain growth in both organic and aqueous environments.^1–7^ These methods allow the precisely regulated polymer synthesis with controlled molecular weights and compositions, thereby enabling fine-tuning of mechanical, thermal, and functional properties. Moreover, they contribute to the creation of advanced materials, including polymers with complex architectures and organic–inorganic hybrid materials.^8–13^

Among these techniques, ATRP utilizes transition metal complexes as catalysts and achieves controlled radical polymerization through reversible redox processes.^13^ In particular, copper-catalyzed ATRP has been widely applied for the polymerization of various monomers.^14,15^ In this system, the halogen atom at the dormant polymer chain end is transferred to the active copper–ligand complex [Cu^I^/L]^+^, generating an active radical and a complex in the higher oxidation state [X–Cu^II^/L]^+^. The active radical propagates the polymer chain, while the growing chain end is reversibly deactivated by reacting with [X–Cu^II^/L]^+^, regenerating [Cu^I^/L]^+^ and forming the dormant halogen-terminated species. The equilibrium between the active and dormant species is strongly shifted toward the dormant state, suppressing the radical concentration and enabling precise control over the polymerization.

However, oxygen severely interferes with radical polymerizations by forming peroxy radicals, thereby trapping the active radicals.^16^ In ATRP, oxygen also oxidizes the active copper complex [Cu^I^/L]^+^, disrupting the catalytic cycle.^17^ Therefore, conventional ATRP is highly oxygen-sensitive and requires tedious deoxygenation prior to polymerization.^18^ To address this limitation, oxygen-tolerant ATRP systems that continuously regenerate Cu^I^ species from Cu^II^ species have been developed, thus maintaining the catalytic activity even in the presence of oxygen.^18–24^ Among these, photoinduced ATRP (photo-ATRP) has attracted considerable attention as an environmentally benign method that enables precise spatiotemporal control.^25–32^ Recent advances include the development of systems with ultralow catalyst concentrations (down to the ppb range)^33^ and efficient polymerizations using red light and near-infrared (NIR) light.^34–39^ Long-wavelength light such as red light and NIR light are particularly promising for practical applications due to their low energy, high biocompatibility, excellent penetration, low scattering, and minimal side reactions.^40,41^ Expanding such efficient controlled radical polymerization techniques to the synthesis of functional materials could lead to the development of versatile functional polymers.

One prominent class of functional soft materials is liquid-crystalline polymers (LCPs).^42^ LCPs possess LC properties, which refers to the combination of the fluidity of liquids and the structural order of crystals, along with the inherent flexibility of polymer chains. They show excellent electrical,^43^ optical,^44–48^ and stimuli-responsive^47–55^ properties based on molecular alignment, and have been applied in a wide range of fields. The LC properties that underlie these functions are strongly influenced by the molecular weight.^56–60^ In addition, the molecular weight distribution of LCPs also influences the exhibition of LC phases.^61,62^ For this reason, controlled radical polymerization, rather than conventional free-radical polymerization, has been widely employed in studies focused on their physical properties and functional performance.^60,63–75^ The ability to efficiently and conveniently synthesize structurally diverse LCPs with controlled molecular weights is expected to accelerate both fundamental understanding and the development of advanced functional materials.

In this study, we demonstrate red-light-driven ATRP to synthesize LCPs, aiming to achieve efficient and precise polymerization under ambient conditions. Well-defined LCPs with high monomer conversions were successfully synthesized under red-light irradiation within several hours in air. Polymer growth was precisely regulated by alternately turning the light on and off, and molecular weights could be modulated by adjusting the polymerization conditions. The synthesized polymers exhibited clear LC properties and large-area, unidirectional molecular alignment on a rubbed substrate. Furthermore, this method was applicable to various mesogenic monomers and polymerizable functional groups, demonstrating the broad versatility of red-light-driven ATRP for LCP synthesis.

## Results and Discussion

### Optimization of Polymerization Conditions

4-[4-(4-Methoxyphenyloxycarbonyl)­oxy]­butyl methacrylate (M4MPB), a monomer previously used in LCP synthesis by conventional ATRP,^76^ was selected as a monomer. Photo-ATRP was conducted in DMSO using a red-light irradiation setup, with ethyl α-bromoisobutyrate (EBiB) as the initiator, [X–Cu^II^/TPMA]^+^ (TPMA: tris­(2-pyridylmethyl)­amine) as the deactivator complex, excess TPMA as the electron donor, and methylene blue (MB^+^) as the organic photocatalyst, following the MB^+^/Cu dual-catalytic system previously reported^34^ (Figure 1). The oxygen tolerance of the red-light-driven ATRP system is attributed to efficient quenching of ground-state oxygen (^3^O_2_) by triplet-excited methylene blue (^3^MB^+*^), leading to the formation of singlet oxygen (^1^O_2_) as the primary reactive oxygen species.^34^ DMSO rapidly reacts with the generated ^1^O_2_ to form dimethyl sulfone, thereby irreversibly consuming reactive oxygen species and effectively suppressing oxygen inhibition under ambient conditions.^28,31,77^ The synthesis conditions and results are summarized in Table 1. The monomer conversion was calculated by ^1^H NMR (Text S1, Figure S1), and the molecular weight and dispersity were evaluated by size exclusion chromatography (SEC) (Figure S2). As a first attempt, the conditions based on a previous report^34^ were adopted as entry 1. Under this condition, polymerization yielded the LCP with a high monomer conversion of 90% after 8 h. SEC analysis showed a monomodal distribution with a dispersity (*D̵*) of 1.27. To further improve the control, polymerization conditions were optimized. In the following experiments, the initial monomer-to-initiator ratio ([M]_0_/[I]_0_) was fixed at 100, and the amounts of other components were indicated as molar equivalents relative to the initiator.

**Figure 1:**
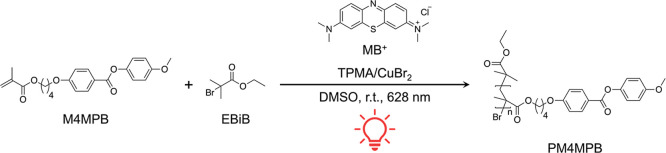
Polymerization scheme for the synthesis of LCP (PM4MPB) by photo-ATRP using red light.

**Table 1:** Synthesis Conditions and Results for the Optimization of Photo-ATRP of M4MPB^a^

entry	EBiB	CuBr_2_	TPMA	MB^+^	time (h)	conv.^b^ (%)	*M*_n,th_ ^c^	*M*_n,app_ ^d^	*D̵* ^d^
1	1	1	2.5	0.025	8	90	34,800	29,700	1.27
2	1	1	1.5	0.025	24	20	7700	11,100	1.15
3	1	1	1.8	0.025	14	78	30,300	24,200	1.20
4	1	1.5	1.8	0.025	12	0	-	-	-
5	1	0.5	1.8	0.025	6	95	36,800	28,900	1.15
6	1	0.5	1.8	0.005	4	90	34,700	27,700	1.14
7	1	0.5	1.8	0.0025	4	89	34,400	26,900	1.13
8	1	0.5	1.8	0.001	12	86	33,300	28,300	1.24

a Reaction conditions: [M4MPB]/[EBiB]/[CuBr_2_]/[TPMA]/[MB^+^] = 100/1/x/y/z; [M4MPB] = 500 mM in DMSO; red-light irradiation (628 nm, 21 mW cm^–2^) in a 1.5 mL vial under ambient atmosphere without degassing.

b Monomer conversion was determined by^1^H NMR spectroscopy.

c Theoretical molecular weight (*M*_n,th_) was calculated as *M*_n,th_ = *M*_EBiB_ + [M]_0_/[I]_0_ × conv./100 × *M*_M4MPB_, where *M*_EBiB_ and *M*_M4MPB_ are the molecular weights of EBiB and M4MPB, respectively.

d Molecular weight (*M*_n,app_) and dispersity (*D̵*) were determined by SEC (THF as eluent) calibrated to polystyrene standards.

First, the effect of the ligand amount was investigated. When the molar equivalent of TPMA was set to 1.5, the monomer conversion significantly dropped to 20% after 24 h (Table 1, entry 2), probably due to insufficient TPMA acting as an electron donor, resulting in greater susceptibility to oxygen inhibition. Increasing the TPMA to 1.8 equiv improved the monomer conversion to 78% in 14 h and reduced the dispersity to 1.20 (Table 1, entry 3). Next, the effect of the amount of copper catalyst was examined. Under the conditions of entry 3, increasing the copper catalyst to 1.5 equiv completely inhibited polymerization (Table 1, entry 4). This behavior can be attributed to the reduced availability of TPMA as an electron donor and heightened oxygen sensitivity. It could also be due to the higher [X–Cu^II^/L]^+^ deactivator concentration shifting the equilibrium away from the active radical state, thereby reducing the concentration of propagating radicals and decreasing the overall polymerization rate. In contrast, reducing the copper catalyst to 0.5 equiv led to efficient and controlled polymerization, achieving a monomer conversion of 95% and *D̵* = 1.15 in 6 h (Table 1, entry 5).

Subsequently, the amount of MB^+^ was optimized. Based on the condition of entry 5, reducing the MB^+^ loading to 0.005 and 0.0025 equiv slightly decreased the dispersity to 1.13 while maintaining high monomer conversions (Table 1, entries 6 and 7). However, further reduction to 0.001 equiv resulted in decreased reaction rate and control (Table 1, entry 8). Therefore, the condition in entry 7, with 0.0025 equiv of MB^+^, a monomer conversion of 89% in 4 h, and *D̵* = 1.13, was identified as the optimal condition. Under this condition, based on the absorption spectrum of MB^+^ (Figure S8), its concentration in the solution, and the vial diameter, it was estimated that approximately 41% of the incident light was absorbed by MB^+^ in the solution (Figure S8). The reaction time under optimized condition is approximately one-fifth of that required for conventional ATRP-based LCP synthesis, demonstrating significantly enhanced efficiency.^60,63,64,76,78^ This significant enhancement in synthetic efficiency is attributed to the efficient scavenging of oxygen and the rapid regeneration of active Cu^I^ species by photoexcited MB^+^, which stabilizes the Cu^I^/Cu^II^ equilibrium during photo-ATRP.^13,34^ In addition to the faster reaction kinetics, this system exhibits excellent oxygen tolerance, enabling well-controlled polymerization under ambient conditions without prior deoxygenation.

### Kinetic Study and Temporal Control

Under the optimized conditions described in the previous section, the kinetics of photo-ATRP were evaluated using samples prepared under the same procedure. At each polymerization time point, aliquots were taken from the reaction solution and analyzed by ^1^H NMR and SEC (Table S1). As the irradiation time increased, the monomodal SEC peak shifted toward higher molecular weights (Figure 2A). ^1^H NMR measurements revealed an induction period of less than 30 min, and linear semilogarithmic kinetic plots were observed, indicating steady consumption of the monomer over time (Figure 2B). Furthermore, the relationship between monomer conversion determined by ^1^H NMR and molecular weights determined by SEC was investigated. The number-average molecular weight exhibited a nearly linear increase with monomer conversion, while the dispersity (*D̵*) was kept below 1.2 over the entire course of polymerization (Figure 2C). These results demonstrate that red-light-driven ATRP offers excellent control not only for amorphous polymers as previously reported but also for the LCPs.

**Figure 2:**
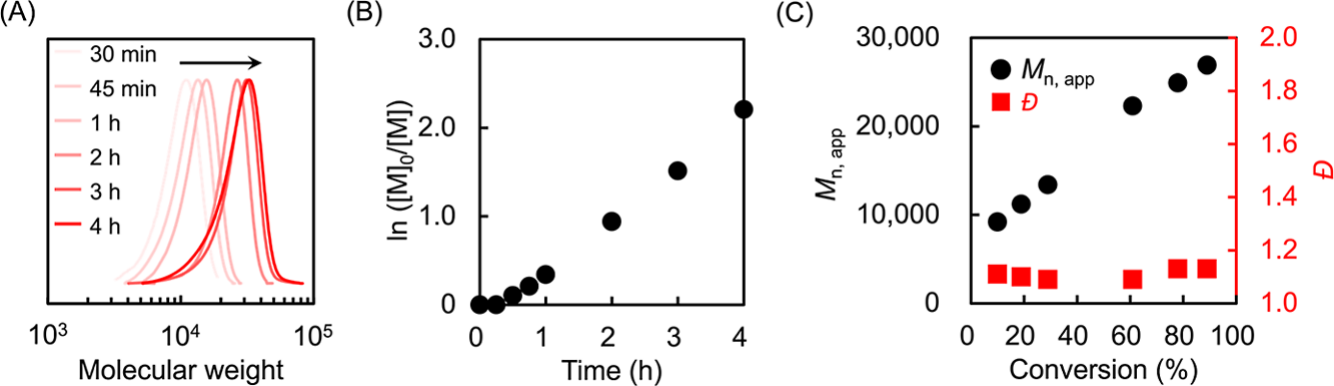
(A) SEC traces evolution with time, (B) first-order kinetic plot, and (C) evolution of molecular weight (*M*_n,app_) and dispersity (*D̵*) with monomer conversion. Reaction conditions: [M4MPB]/[EBiB]/[CuBr_2_]/[TPMA]/[MB^+^] = 100/1/0.5/1.8/0.0025, [M4MPB] = 500 mM.

Additionally, the light-mediated control of the polymer growth was examined by periodically interrupting and resuming light irradiation (Figure 3, Table S2). When the light was turned off, no polymerization occurred, whereas upon irradiation, MB^+^/Cu catalyst effectively scavenged dissolved oxygen and regenerated the active Cu^I^/ligand complex, allowing polymerization to resume. When the red light was repeatedly turned on and off, polymerization correspondingly stopped and restarted, confirming the reversible nature of the light-mediated process and its excellent spatiotemporal controllability. These findings clearly demonstrate that the degree of polymer growth in red-light-driven ATRP can be precisely controlled in air by light irradiation.

**Figure 3:**
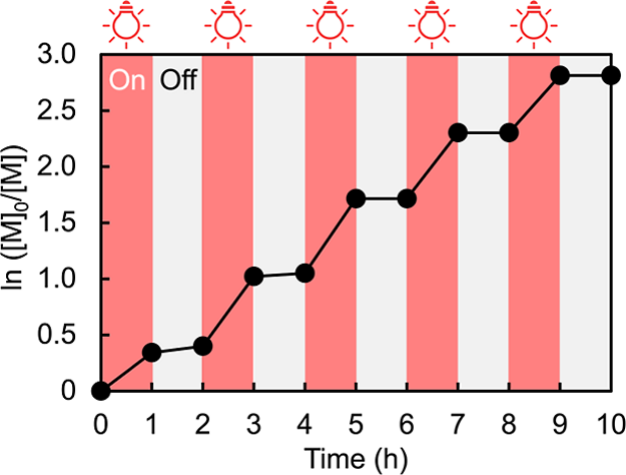
Temporal control of PM4MPB synthesis by switching the red light on and off.

### Modulation of Molecular Weight

The LC property of LCPs is known to be highly dependent on molecular weight. To investigate this effect, LCPs with different molecular weights were synthesized by red-light-driven ATRP (Table 2). Based on the optimized conditions, the monomer concentration was fixed, and [M]_0_/[I]_0_ was varied from 12.5 to 200 in five steps by adjusting the initiator concentration. In all cases, high monomer conversions of 70–90% were achieved within 4 h. SEC analysis showed that the monomodal peaks shifted toward higher molecular weights with increasing [M]_0_/[I]_0_ (Figure 4). The lower monomer conversion observed at [M]_0_/[I]_0_ = 12.5 is attributed to a deviation from the optimal polymerization conditions caused by changes in the relative concentrations of the catalyst and ligand with respect to the radical concentration. In addition, discrepancies between the molecular weights determined by SEC (*M*_n,app_) and the theoretical values (*M*_n,th_) were observed depending on the polymerization conditions. Such discrepancies are commonly reported for LCPs and arise from the fact that SEC provides relative molecular weight values calibrated against polystyrene standards, while LCPs possess distinct molecular architectures and conformational characteristics compared to polystyrene.^63,67^ Nevertheless, the dispersity remained below 1.16 across all polymerization conditions, indicating that controlled polymerization was maintained. These results demonstrate that red-light-driven ATRP enables facile synthesis of LCPs with narrow molecular weight distributions and tunable molecular weights by simply varying the [M]_0_/[I]_0_ ratio.

**Table 2:** Photo-ATRP of M4MPB with Varied Monomer-To-Initiator Ratios to Target Different Molecular Weights^a^

entry	[M]_0_/[I]_0_	[EBiB] (mM)	conv.^b^ (%)	*M*_n,th_ ^b^	*M*_n,app_ ^b^	*D̵* ^b^
1	12.5	40	71	3600	7800	1.14
2	25	20	80	8000	12,000	1.14
3	50	10	87	16,900	17,700	1.13
4	100	5	89	34,400	26,900	1.13
5	200	2.5	86	66,200	49,300	1.16

a Reaction conditions: [M4MPB] = 500 mM; [EBiB] = 2.5–40.0 mM; [CuBr_2_] = 2.5 mM; [TPMA] = 9.0 mM; [MB^+^] = 12.5 μM in DMSO; red-light irradiation (628 nm, 21 mW cm^–2^) for 4 h in a 1.5 mL vial under ambient atmosphere without degassing.

b Data obtained as described for Table 1.

**Figure 4:**
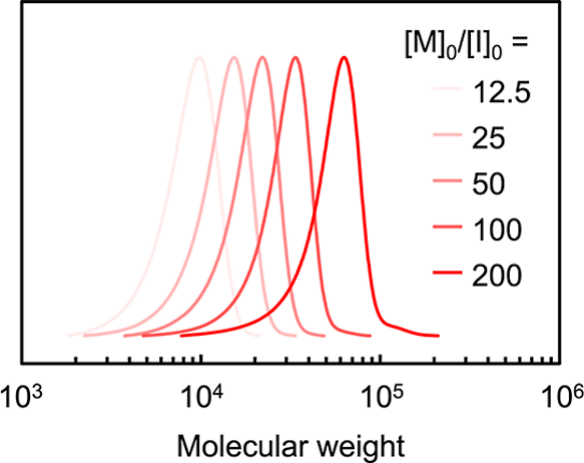
SEC traces of PM4MPB synthesized with different target molecular weights.

### Molecular Weight Dependence of LC Properties

The LC properties of PM4MPB synthesized by red-light-driven ATRP were evaluated using differential scanning calorimetry (DSC) with the observation of LC textures by polarized optical microscopy (POM) (Figure 5 and Table 3). During the heating process in DSC measurements, all samples with different molecular weights exhibited a baseline shift around 50 °C corresponding to the glass transition and an endothermic peak near 105 °C associated with phase transition (Figure 5A). POM observations conducted around the endothermic transition temperature revealed Schlieren textures characteristic of a nematic phase below the transition temperature, while dark images were observed above it (Figure 5B). These behaviors are consistent with the properties of PM4MPB previously synthesized by conventional ATRP,^76^ confirming that the polymers obtained by red-light-driven ATRP also exhibit a nematic LC phase. Furthermore, a plot of transition temperatures against molecular weight showed that both the glass transition and the phase transition temperatures decreased as molecular weight decreased (Figure 5C), which is in agreement with previously reported trends for LCPs.^56^ Since control of these transition temperatures is important in the development of LCPs, the ability of this method to synthesize LCPs with tunable molecular weights under ambient conditions will play a significant role in advancing both fundamental studies and applications of LCPs.

**Figure 5:**
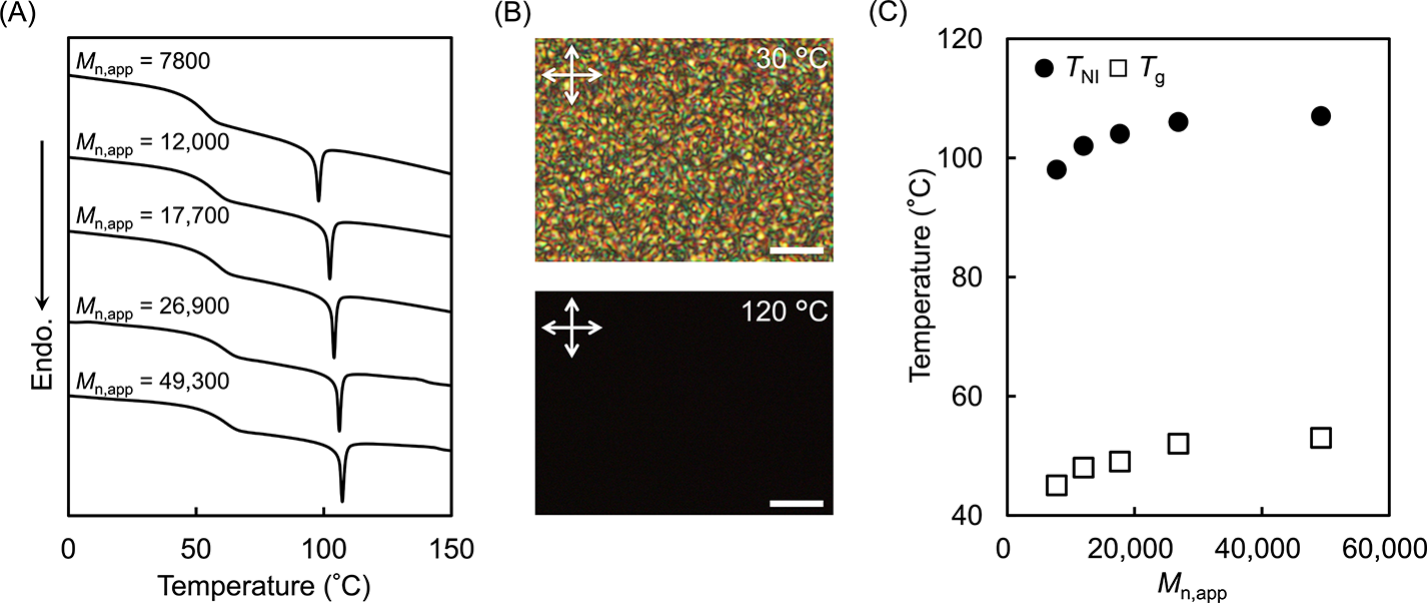
(A) DSC thermograms of PM4MPB with different molecular weights on the third heating at a scanning rate of 10 °C min^–1^. (B) POM images of PM4MPB (*M*_n,app_ = 26,900) observed during heating. White arrows indicate the direction of crossed polarizers. Scale bars: 10 μm. (C) *T*_NI_ (closed circle) and *T*_g_ (open square) as a function of *M*_n,app_.

**Table 3:** Thermodynamic Properties of PM4MPB Samples with Varying Molecular Weights Evaluated by DSC During the Third Heating Cycle

*M*_n,app_	*T*_g_ (°C)	*T*_NI_ (°C)	Δ*H*_NI_ (kJ mol^–1^)
7800	45	98	0.5
12,000	48	102	0.6
17,700	49	104	0.6
26,900	52	106	0.6
49,300	53	107	0.6

Next, the molecular alignment behavior of PM4MPB films with different molecular weights were investigated. Thin films were prepared on rubbed quartz substrates covered with a polyimide coating and characterized using POM and polarized UV–vis absorption spectroscopy. As a representative example, the results for a sample with *M*_n,app_ = 26,900 are shown in Figure 6. Under crossed polarizers, the film exhibited alternating bright and dark images by every 45° of rotation, appearing dark when the polarizers were set parallel or perpendicular to the rubbing direction (Figure 6A). In the polarized UV–vis absorption spectra, the absorbance for light polarized along the rubbing direction was higher than that for light polarized perpendicular to it at the absorption band attributed to the mesogenic moiety (Figure 6B). Similar results were obtained for LCPs with other molecular weights (Figures S3 and S4). These findings indicate that the mesogenic units are uniformly aligned along the rubbing direction across all films, regardless of molecular weight.

**Figure 6:**
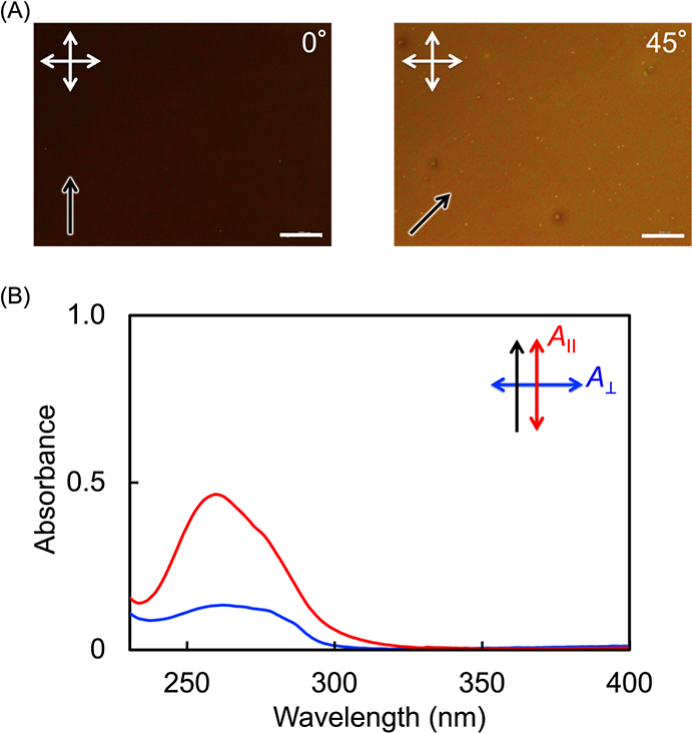
(A) POM images of a PM4MPB thin film (*M*_n,app_ = 26,900) spin-coated and annealed on a rubbed alignment layer. Black arrows indicate rubbing direction, and white crossed arrows indicate the polarization directions. Scale bars: 200 μm. (B) Polarized UV–vis absorption spectra of the same film measured with light polarized parallel (red) and perpendicular (blue) to the rubbing direction.

To quantify the degree of molecular alignment, the birefringence (Δ*n*) was measured using a Berek compensator, and the order parameter (*S*) was calculated from the polarized UV–vis absorption spectra. The resulting values of Δ*n* and *S*, together with the film thickness (*d*), are summarized in Table 4. *S* was determined at 257 nm, the maximum absorption wavelength, using the equation *S* = (*A*_∥_ – *A*_⊥_)/(*A*_∥_ + 2*A*_⊥_), where *A*_∥_ and *A*_⊥_ are the absorbance values parallel and perpendicular to the rubbing direction, respectively. The values of *S* were nearly constant regardless of molecular weight or mesogenic structure. This consistency is attributed to the thinness of the films (approximately 60 nm), which allowed the alignment force from the substrate to dominate molecular alignment.

**Table 4:** Characterization of Thin Films of PM4MPB with Varying Molecular Weights

*M*_n,app_	*d* (nm)	Δ*n*	*S*
7800	59	0.10	0.48
12,000	62	0.10	0.48
17,700	60	0.09	0.47
26,900	61	0.09	0.46
49,300	58	0.10	0.47

### Application of Photo-ATRP to the Synthesis of Various LC Polymers

The LC property, molecular alignment, and their resulting physical and functional characteristics are strongly influenced by the type of polymerizable group and the structure of the mesogenic moiety. To extend the versatility of red-light-driven ATRP for LCP synthesis, three structurally different LCPs were synthesized. Specifically, methacrylate-type monomers bearing cyano-terminated phenyl benzoate (M6CPB), and acrylate-type monomers with methoxy-terminated phenyl benzoate (A6MPB) or cyanobiphenyl (A6CB) mesogenic side chains were selected (Figure 7). The polymerization results are summarized in Table 5.

**Figure 7:**
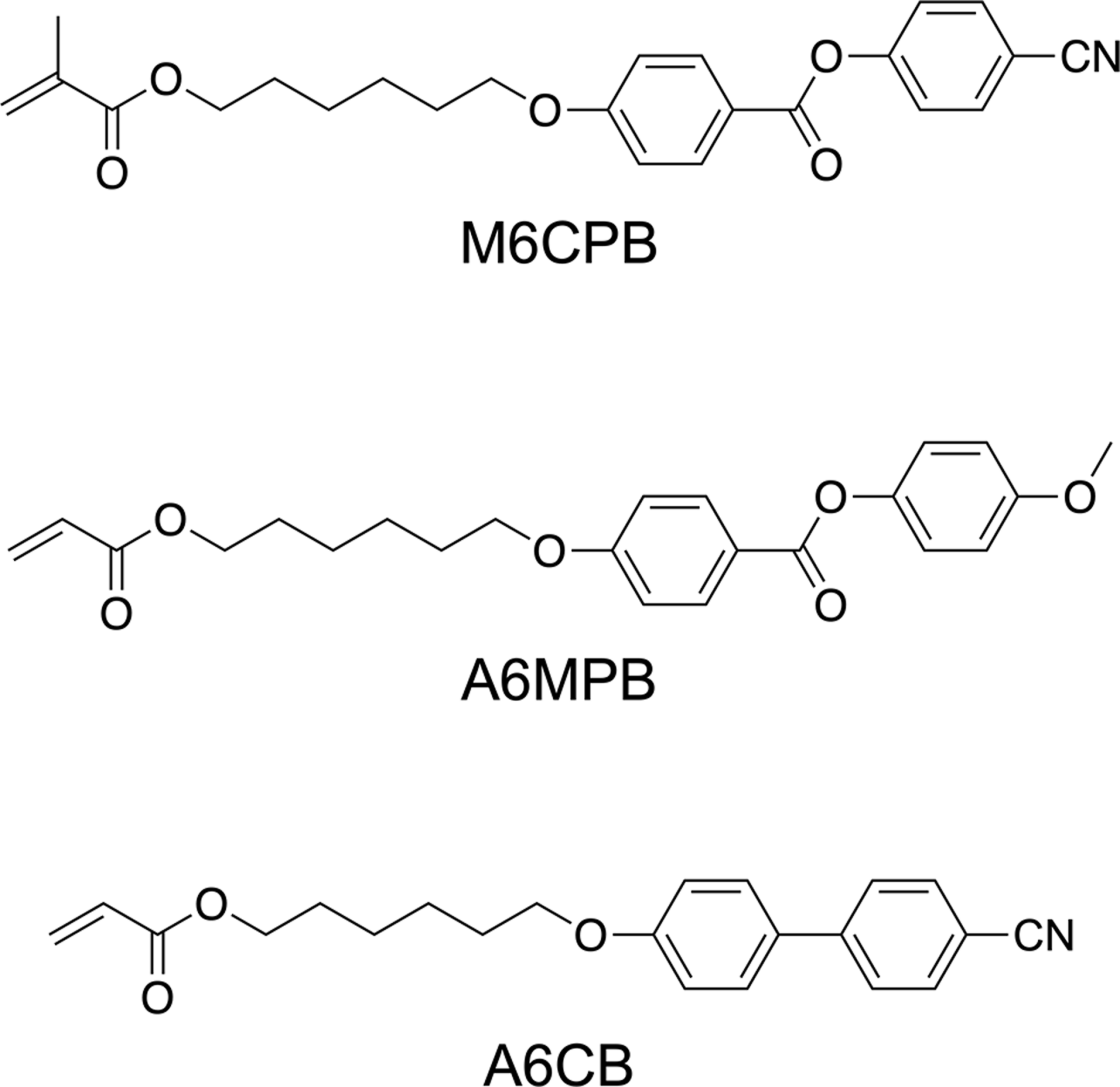
Chemical structures of monomers used for photo-ATRP.

**Table 5:** Synthesis Results of Various LCPs by Photo-ATRP^a^

monomer	ligand	conv.^b^ (%)	*M*_n,th_ ^c^	*M*_n,app_ ^b^	*D̵* ^b^
M6CPB	TPMA	82	33,400	29,700	1.12
A6MPB	Me_6_TREN	78	31,200	28,100	1.19
A6CB	Me_6_TREN	62	21,600	27,400	1.07

a Reaction conditions: [monomer] = 500 mM; [EBiB] = 5.0 mM; [CuBr_2_] = 2.5 mM; [ligand] = 9.0 mM; [MB^+^] = 12.5 μM in DMSO; red-light irradiation (628 nm, 21 mW cm^–2^) for 3 h in a 1.5 mL vial under ambient atmosphere without degassing.

b Determined as described for Table 1.

c Theoretical molecular weight (*M*_n,th_) was calculated as described for Table 1, except that the molecular weight of each corresponding monomer was used in place of M4MPB.

Polymerizations of the methacrylate monomer was conducted under the same conditions as those optimized for M4MPB (Table 1, entry 7). Efficient polymerization was achieved regardless of the mesogenic moiety structure, with M6CPB yielding 82% monomer conversion and a dispersity (*D̵*) of 1.12 after 3 h of red-light irradiation. By comparison, the acrylate monomer A6MPB exhibited slow polymerization under identical conditions, giving only 5% monomer conversion after 4 h (Table S3, Figure S5). Under these polymerization conditions, even after 24 h of light irradiation, the monomer conversion remained below 5%, indicating that the low conversion was not simply due to a slow polymerization rate. Therefore, we changed the ligand from TPMA to Me_6_TREN, which possesses a higher ATRP equilibrium constant (*K*_ATRP_).^8,13^ The molar ratios were kept consistent as [Monomer]/[EBiB]/[CuBr_2_]/[Me_6_TREN]/[MB^+^] = 100/1/0.5/1.8/0.0025, with only the ligand substituted. Under the modified condition, A6MPB reached 78% monomer conversion with *D̵* = 1.19 after 3 h. A6CB also underwent successful polymerization under the same condition, resulting in 62% monomer conversion and *D̵* = 1.09. Although the effect of monomer structure on red-light-driven ATRP has not been systematically studied, we consider that the decreased conversion observed in the polymerization of A6CB is due to the polarity of the cyanobiphenyl group, which likely perturbs the coordination environment of the copper catalyst.

These results indicate that red-light-driven ATRP can be effectively applied to the synthesis of LCPs with various terminal polarities and backbone structures, demonstrating the broad applicability of this method.

### Controlled Synthesis of LC Polymers under Diverse Light Sources

Methylene blue (MB^+^), the photocatalyst used in this study, has a broad absorption range covering from UV to NIR light.^34^ This broad absorption profile suggests that the photo-ATRP system may tolerate variations in irradiation conditions for synthesizing LCPs. To evaluate this tolerance, polymerizations were conducted under different light conditions, including green-light irradiation and red-light irradiation at reduced intensity (Table 6, Figure S7). The polymerization mixtures were prepared under the optimized conditions (Table 1, entry 7) and placed in 1.5 mL vials. Upon irradiation with green light (518 nm, 50 mW cm^–2^), the synthesis of an LCP with a narrow molecular weight distribution was successfully achieved (conv. = 41%, *M*_n,app_ = 15,300, *D̵* = 1.16). Despite the higher light intensity compared to red-light irradiation, the monomer conversion was lower. This behavior can be attributed to the significantly lower absorbance of MB^+^ at 518 nm relative to 628 nm, which led to a reduced generation of catalytically active Cu^I^ species via photoinduced electron transfer. Consistently, when the light intensity was reduced to 10 mW cm^–2^ under red light (628 nm) irradiation, the monomer conversion also decreased while maintaining a comparable molecular weight distribution (conv. = 71%, *M*_n,app_ = 20,700, *D̵* = 1.15). Taken together, these results demonstrate that the photo-ATRP system tolerates variations in irradiation wavelength and light intensity while maintaining controlled polymerization behavior.

**Table 6:** Photo-ATRP of M4MPB Performed Under Green Light, Red Light at Low Intensity, Sunlight, and Smartphone Flashlight^a^

light source	conv.^b^ (%)	*M*_n,th_ ^b^	*M*_n,app_ ^b^	*D̵* ^b^
green LED[Table-fn t6fn3]	41	15,800	15,300	1.16
red LED[Table-fn t6fn4]	71	27,100	20,700	1.15
sun^e^	82	31,700	25,000	1.17
smartphone^f^	84	32,300	24,700	1.14

a Reaction conditions: [M4MPB] = 500 mM; [EBiB] = 5.0 mM; [CuBr_2_] = 2.5 mM; [TPMA] = 9.0 mM; [MB^+^] = 12.5 μM in DMSO; irradiated for 4 h in a 1.5 mL vial under ambient atmosphere without degassing.

b Determined as described for Table 1.

e Light intensity at 518 nm: 50 mW cm^–2^.

f Light intensity at 628 nm: 10 mW cm^–2^.

g Light intensity at 545 nm, measured using a band-pass filter centered at 545 nm with a full width at half-maximum (fwhm) of 10 nm and an infrared cut filter: approximately 1 mW cm^–2^.

h Light intensity at 545 nm, measured using a 545 nm band-pass filter (fwhm 10 nm): 40 mW cm^–2^.

To further demonstrate the applicability to practical irradiation environments, polymerizations were conducted using sunlight and a more accessible light source such as the LED flashlight of a smartphone (Table 6, Figure S7). When exposed to sunlight for 4 h, the reaction achieved a monomer conversion of 82%, *M*_n,app_ = 25,000, and *D̵* = 1.17, confirming the successful controlled synthesis of the LCP using sunlight. Since the spectrum and intensity of sunlight vary depending on the season and time of day, reproducibility can be improved by monitoring the effective irradiation intensity at the relevant wavelength and adjusting the irradiation time accordingly. Similarly, irradiation with a smartphone flashlight for 4 h led to 84% conversion, *M*_n,app_ = 24,700, and *D̵* = 1.14, demonstrating that controlled synthesis is also achievable using smartphone LED flashlight. These results indicate that controlled synthesis of LCPs can be performed using readily accessible light sources, without the need for dedicated irradiation equipment.

## Conclusion

In this study, red-light-driven ATRP using an organic photocatalyst was employed to synthesize LCPs, a class of functional polymers. The method enabled efficient polymerization with well-controlled molecular weight distributions under ambient conditions, eliminating the need for deoxygenation. A high monomer conversion (∼90%) was achieved within a few hours, significantly reducing reaction time compared to conventional methods. The polymerization was precisely controlled by modulating irradiation time or switching the red light on and off. By adjusting the initiator concentration, LCPs with various molecular weights and narrow molecular weight distributions were obtained, exhibiting LC properties and molecular alignment behavior comparable to those of conventionally synthesized LCPs. The method was also applicable to a wide range of mesogenic monomers and polymerizable groups, demonstrating its versatility. Furthermore, polymerization was successfully performed using common light sources such as sunlight and smartphone LED flashlight. Additionally, we anticipate that the appropriate selection of photosensitizers, including those responsive to longer wavelengths, will enable the future development of precision syntheses of LC polymers, such as systems incorporating monomers bearing dyes that absorb the visible light. Overall, this approach offers a powerful platform for the efficient synthesis of LCPs and is expected to accelerate both the development of functional soft materials and the elucidation of their structure–property relationships.

## Materials and Methods

### Materials

The monomers 4-[4-(4-methoxyphenyloxycarbonyl)­oxy]­butyl methacrylate (M4MPB) and 6-[4-(4′-cyanobiphenyl)­oxy]­hexyl acrylate (A6CB) were purchased from Sundia MediTech Company (Shanghai, China), and Chemfish Tokyo Co., Ltd. (Japan), respectively, whereas 6-[4-(4-cyanophenyloxycarbonyl)­oxy]­hexyl methacrylate (M6CPB) and 6-[4-(4-methoxyphenyloxycarbonyl)­oxy]­hexyl methacrylate (A6MPB) were kindly provided by ENEOS Corporation (Japan). All monomers were purified by recrystallization from ethanol before use.

Ethyl 2-bromoisobutyrate (EBiB) and tris­(2-pyridylmethyl)­amine (TPMA) were obtained from Tokyo Chemical Industry Co., Ltd. (Tokyo, Japan). Tris­[2-(dimethylamino)­ethyl]­amine (Me_6_TREN), copper­(II) bromide (CuBr_2_), and dimethyl sulfoxide (DMSO) were obtained from FUJIFILM Wako Pure Chemical Corp. (Tokyo, Japan). Methylene blue (MB^+^) was purchased from Sigma-Aldrich (Tokyo, Japan). All reagents except the monomers were used as received.

### Synthesis of LC Polymers by Photo-ATRP

Stock solutions of CuBr_2_ (187.5 mM in DMSO), TPMA (337.5 mM in DMSO), EBiB (250 mM in DMSO), and MB^+^ (937.5 μM in DMSO) were prepared. A typical procedure for the synthesis of PM4MPB is described below. The photo-ATRP solution (1.5 mL) was prepared by mixing M4MPB (288.3 mg), DMSO (1.39 mL), and the stock solutions of CuBr_2_ (20 μL), TPMA (40 μL), EBiB (30 μL), and MB^+^ (20 μL). The final concentrations were as follows: M4MPB, 500 mM; CuBr_2_, 2.5 mM; TPMA, 9.0 mM; EBiB, 5.0 mM; and MB^+^, 12.5 μM. The resulting solution was transferred to a 1.5 mL vial, sealed under ambient atmosphere, and placed in a plastic container (diameter = 5 cm, height = 7 cm) equipped with red LED strips (628 nm, 10 or 21 mW cm^–2^) or green LED stripes (518 nm, 50 mW cm^–2^) and a cooling fan to maintain room temperature. For the demonstration of photo-ATRP using a smartphone, the LED flashlight of a smartphone was used as a light source. The emission spectra of the red LED, green LED, and smartphone LED flashlight are shown in Figure S8. The solution was stirred at 500 rpm and irradiated for the designated duration. After the reaction, the polymer solution was analyzed by ^1^H NMR and SEC.

For kinetic and temporal control experiments, aliquots were withdrawn at specific time intervals using a syringe. The reaction mixtures were passed through a basic alumina column using chloroform as the eluent. The product was purified by evaporating the solvent, followed by three cycles of dissolution in chloroform, precipitation with hexane, and centrifugation to afford the polymer.

### Film Preparation

Aligned thin films of LCPs were prepared on synthetic quartz substrates as follows. The substrates were sequentially cleaned by ultrasonication in a neutral detergent, ultrapure water twice, 2-propanol, followed by drying and exposure to UV–ozone (NL-UV42, Nippon Laser & Electronics Lab Co., Ltd., Nagoya, Japan). The alignment layer was prepared by spin-coating a precursor solution (AL-1254, JSR Corp., Tokyo, Japan) onto the cleaned substrates using a spin coater (MS-B100, Mikasa Co., Ltd., Tokyo, Japan), followed by sequential annealing at 60 °C for 1 min and 220 °C for 1 h. The resulting alignment layer was rubbed using a rubbing equipment (MRG-100, EHC Co., Ltd., Tokyo, Japan). A 1 wt % solution of PM4MPB in 1,1,2-trichloroethane was spin-coated onto the rubbed substrates and annealed at 120 °C for 60 min. The samples were then cooled gradually to 60 °C at a rate of 0.5 °C min^–1^, followed by cooling to room temperature at 10 °C min^–1^.

### Characterization

The progress of polymerization and monomer conversion were evaluated by ^1^H NMR spectroscopy using an Avance III 400 MHz spectrometer (Bruker BioSpin, Germany). Molecular weights and molecular weight distributions were determined by size-exclusion chromatography (SEC) calibrated to polystyrene standards using tetrahydrofuran (THF) as eluent. The SEC system was equipped with a Shodex LF-804 column and an RI-101 refractive index detector (Resonac Corp., Tokyo, Japan). Differential scanning calorimetry (DSC) was conducted using an Exstar DSC7020 instrument (Hitachi High-Tech Analysis Corp., Tokyo, Japan). Polarized optical microscopy (POM) images were taken using a microscope (BX50, Olympus Corp., Tokyo, Japan) equipped with a hot stage (HS82, Mettler-Toledo, Switzerland). A Berek compensator (U-CBE, Olympus Corp., Tokyo, Japan) was attached to the microscope for the determination of the birefringence. Polarized ultraviolet–visible (UV–vis) absorption spectroscopy was performed using a spectrophotometer (V-670, JASCO Corp., Hachioji, Japan). Film thicknesses were determined using a contact profiler (ET4000A, Kosaka Laboratory Ltd., Tokyo, Japan).

## Supplementary Material



## Notes

Calculation of the monomer conversion by ^1^H NMR spectroscopy; SEC traces of PM4MPB samples polymerized under various conditions; kinetic data for PM4MPB synthesis by photo-ATRP; data for temporal control M4MPB polymerization by switching the red light on and off; POM images and polarized UV–vis absorption spectra of PM4MPB thin films spin-coated and annealed on rubbed alignment layers; photo-ATRP result of A6MPB using TPMA as ligand; SEC traces of LCPs synthesized using different monomers; SEC traces of PM4MPB synthesized under green light, red light at low intensity, sunlight, and smartphone flashlight; emission spectra of the red LED, green LED, and smartphone LED flashlight used as light sources for photo-ATRP, and the UV–vis absorption spectrum of MB^+^ (PDF)
