# Supplementary material for: Controlled Synthesis of Liquid-Crystalline Polymers Under Ambient Conditions by Red-Light-Driven ATRP
Source: Macromolecules. 2026 Feb 16;59(5):2933–42. doi: 10.1021/acs.macromol.5c03040 (PMC12981020; doi:10.1021/acs.macromol.5c03040)
Supplement: Supplementary file 1 [file ma5c03040_si_001.pdf]

Supporting Information for

Controlled Synthesis of Liquid-Crystalline Polymers under Ambient Conditions by Red-Light-Driven ATRP

Kaito Takahashi,[a,b] Kaho Nakano,[a,b] Xiaolei Hu,[c] Khidong Kim,[c] Hironobu Murata,[c] Krzysztof Matyjaszewski,[c]* Atsushi Shishido,[a,b,d]* and Shoichi Kubo[a,b]*

[a]Laboratory for Chemistry and Life Science, Institute of Integrated Research, Institute of Science Tokyo, Yokohama 226-8501, Japan

[b]Department of Chemical Science and Engineering, School of Materials and Chemical Technology, Institute of Science Tokyo, Meguro, Tokyo 152-8552, Japan

[c]Department of Chemistry, Carnegie Mellon University, Pittsburgh, Pennsylvania 15213, United States

[d]Research Center for Autonomous Systems Materialogy, Institute of Integrated Research, Institute of Science Tokyo, Yokohama 226-8501, Japan

***E-mail**: km3b@andrew.cmu.edu, ashishid@res.titech.ac.jp, kubo@res.titech.ac.jp

---

This file includes:

Text S1. Calculation of the monomer conversion.

Figure S1. Representative ^{1}H NMR spectrum of crude PM4MPB.

Figure S2. SEC traces of PM4MPB samples polymerized under various conditions.

Table S1. Kinetic data for PM4MPB synthesis by photo-ATRP.

Table S2. Temporal control of M4MPB polymerization by switching the red light on and off.

Figure S3. POM images of PM4MPB thin films spin-coated and annealed on rubbed alignment layers.

Figure S4. Polarized UV−vis absorption spectra of PM4MPB thin films spin-coated and annealed on rubbed alignment layers.

Table S3. Photo-ATRP of A6MPB using TPMA as ligand.

Figure S5. SEC trace of A6MPB after polymerization using TPMA as ligand.

Figure S6. SEC traces of LC polymers synthesized using different monomers.

Figure S7. SEC traces of PM4MPB synthesized under green light, red light (10 mW cm^{-2}), sunlight, and smartphone flashlight.

Figure S8. Emission spectra of the red LED, green LED, and smartphone LED flashlight used as light sources for photo-ATRP, together with the UV−vis absorption spectrum of MB^+.

Text S1. Calculation of the Monomer Conversion.

The monomer conversion was determined from the ^{1}H NMR spectra of the crude reaction mixtures using Equation S1. The calculation was based on the integral at 8.1 ppm ($I_{8.1}$), corresponding to two equivalent aromatic protons present in both monomer and polymer, and the integral at 6.1 ppm ($I_{6.1}$), corresponding to the vinyl protons of the methacrylate group, present only in the monomer:

$$\text{Conv.} = \frac{I_{8.1\ \text{ppm}} - I_{6.1\ \text{ppm}} \times 2}{I_{8.1\ \text{ppm}}} \times 100 \qquad \textbf{Eq. S1}$$

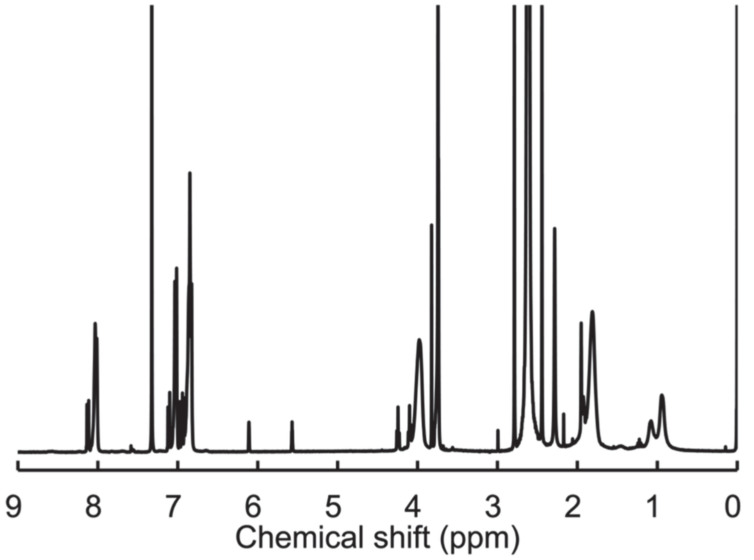

Figure S1. Representative ^{1}H NMR spectrum of crude PM4MPB (Table 1, entry 7).

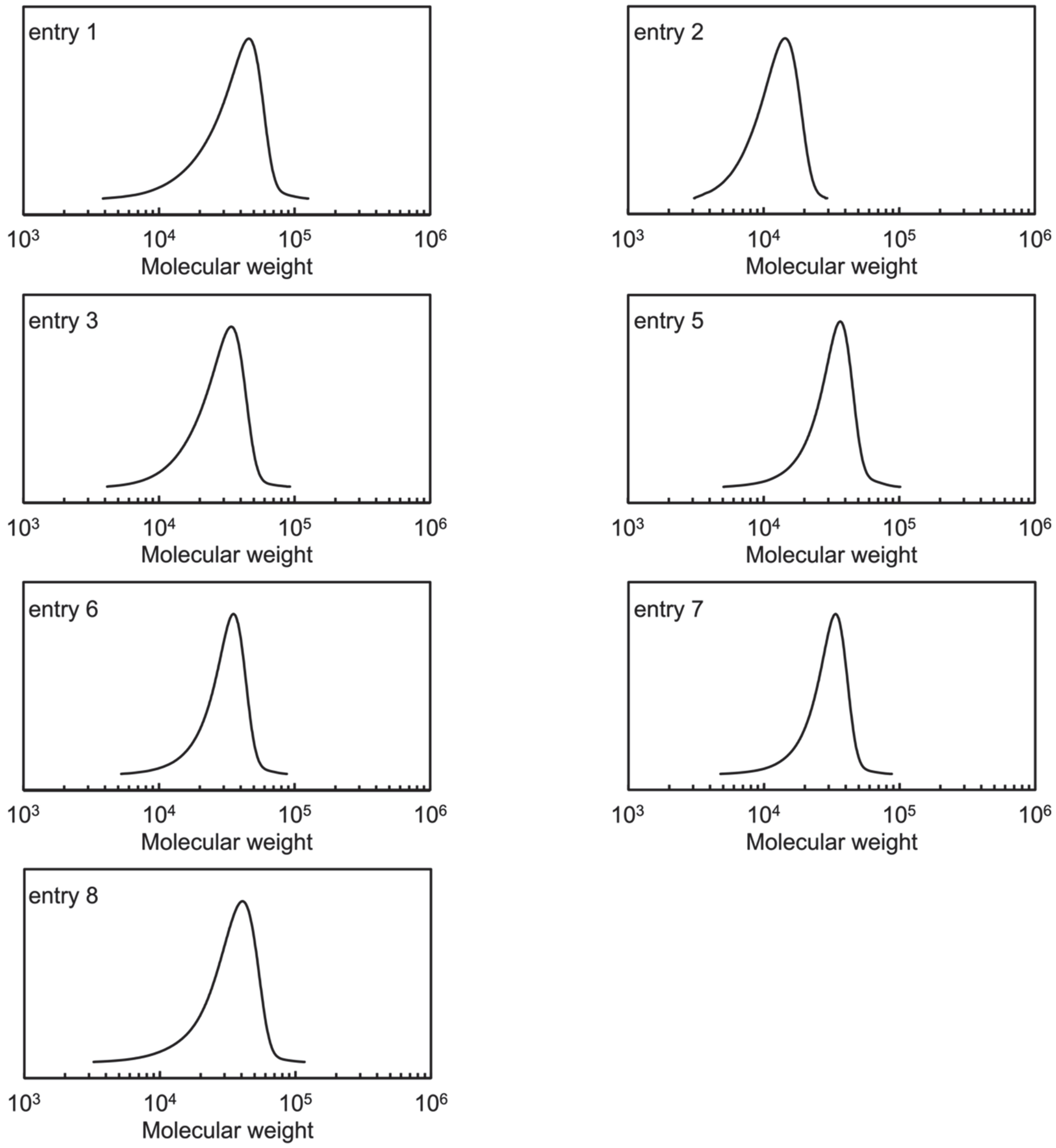

Figure S2. SEC traces of PM4MPB samples polymerized under various conditions (Table 1).

Table S1. Kinetic Data for PM4MPB Synthesis by Photo-ATRP[a]

time (h)	conv.[b] (%)	$M_{n,app}$[c]	$Đ$[c]
0	0	-	-
0.25	0	-	-
0.5	10	9200	1.11
0.75	19	11,200	1.10
1	29	13,400	1.09
2	61	22,300	1.09
3	78	24,900	1.13
4	89	26,900	1.13

[a]Reaction conditions: [M4MPB]/[EBiB]/[$CuBr_2$]/[TPMA]/[MB^+] = 100/1/0.5/1.8/0.0025; [M4MPB] = 500 mM in DMSO; red-light irradiation (628 nm, 21 mW cm^{-2}) in a 1.5 mL vial under ambient atmosphere without degassing. [b]Monomer conversion was determined by ^1H NMR spectroscopy. [c]Molecular weight ($M_{n,app}$) and dispersity ($Đ$) were determined by SEC (THF as eluent) calibrated to polystyrene standards.

Table S2. Temporal Control of M4MPB Polymerization by Switching the Red Light On and Off[a]

time (h)	light	conv.[b] (%)
0	off	0
0–1	on	29
1–2	off	33
2–3	on	64
3–4	off	65
4–5	on	82
5–6	off	82
6–7	on	90
7–8	off	90
8–9	on	94
9–10	off	94

[a]Reaction conditions: [M4MPB]/[EBiB]/[$CuBr_2$]/[TPMA]/[MB^+] = 100/1/0.5/1.8/0.0025; [M4MPB] = 500 mM in DMSO; red-light irradiation (628 nm, 21 mW cm^{-2}) in a 1.5 mL vial under ambient atmosphere without degassing.

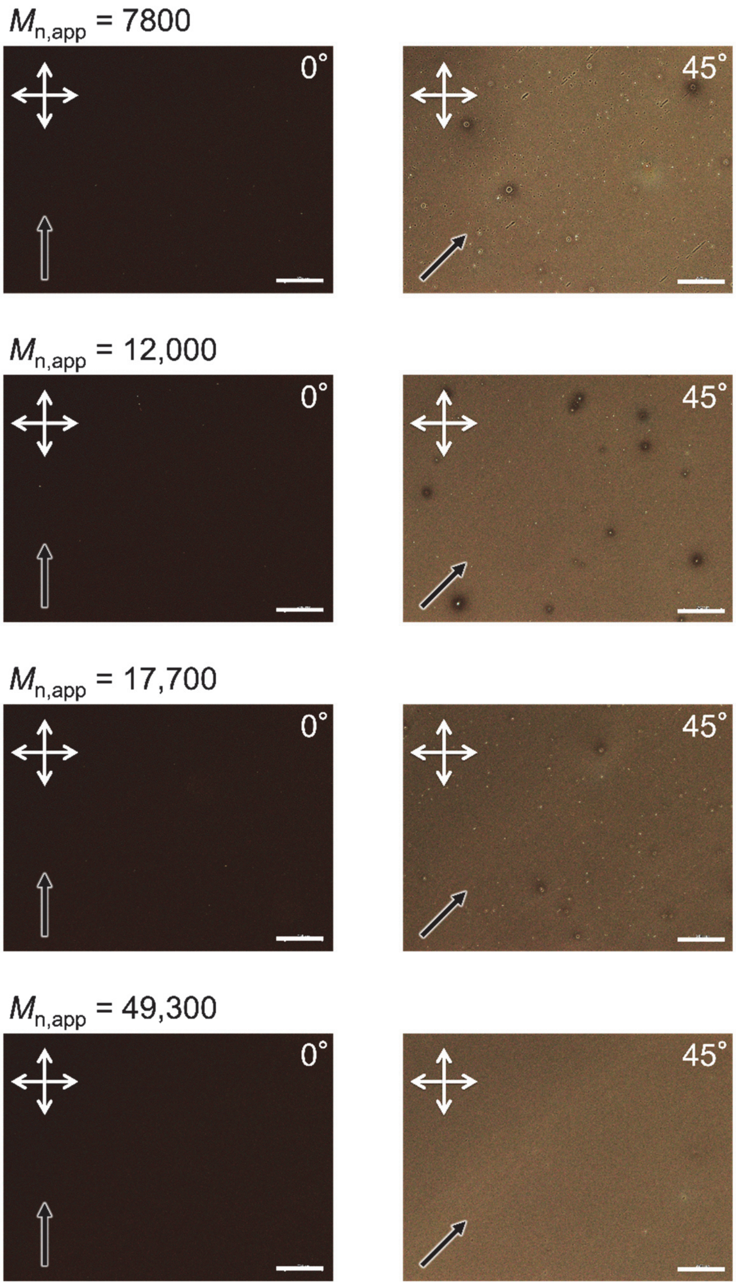

Figure S3. POM images of PM4MPB thin films spin-coated and annealed on rubbed alignment layers. White crossed arrows indicate the polarization directions, and yellow arrows indicate the rubbing directions. Scale bars, 200 µm.

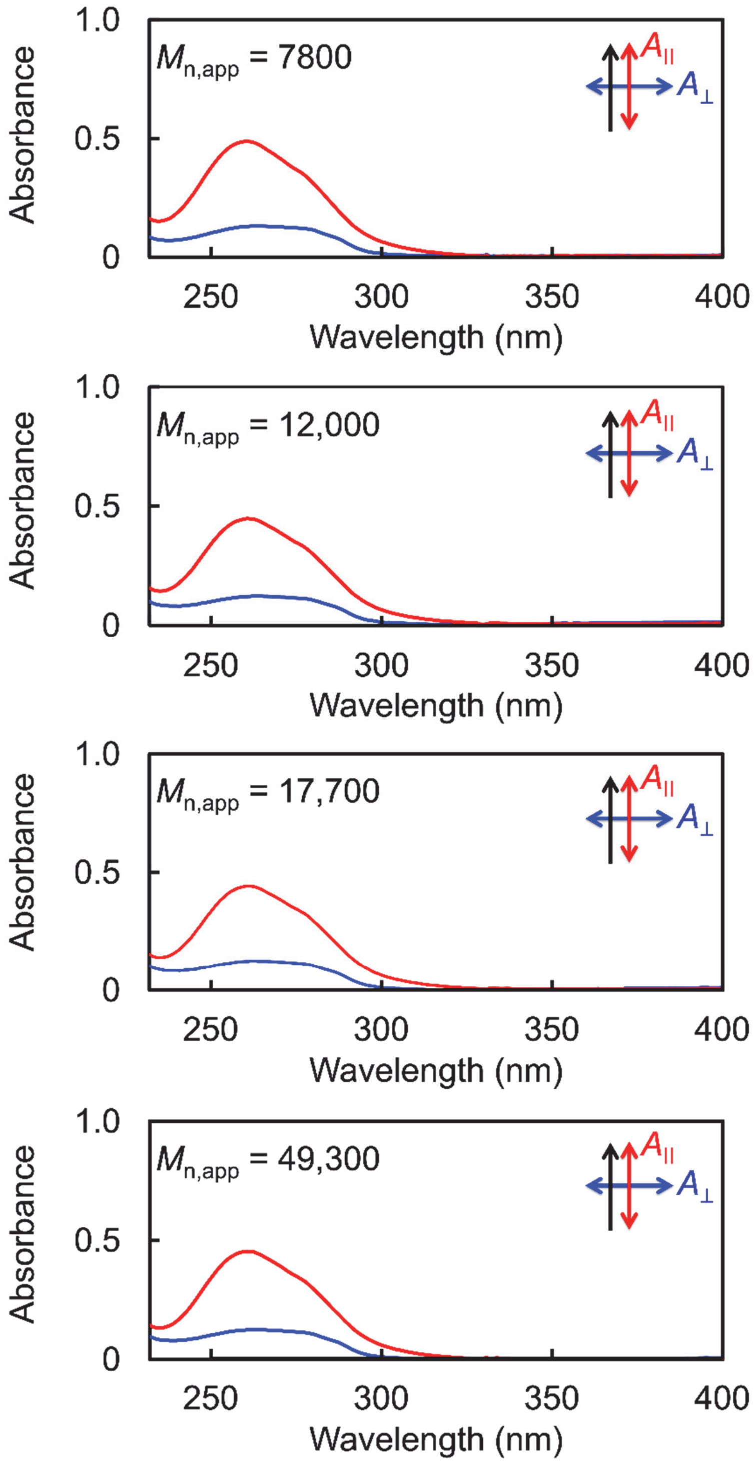

Figure S4. Polarized UV−vis absorption spectra of PM4MPB thin films spin-coated and annealed on rubbed alignment layers. Red and blue lines represent absorbance parallel and perpendicular to the rubbing direction, respectively. Black arrows indicate the rubbing directions.

Table S3. Photo-ATRP of A6MPB Using TPMA as Ligand[a]

ligand	conv.[b] (%)	$M_{n,th}$	$M_{n,app}$[c]	$Đ$[c]
TPMA	5	2400	3900	1.07

[a]Reaction conditions: [A6MPB]/[EBiB]/[$CuBr_2$]/[TPMA]/[MB^+] = 100/1/0.5/1.8/0.0025; [A6MPB] = 500 mM in DMSO; red-light irradiation (628 nm, 21 mW cm^{-2}) for 4 h in a 1.5 mL vial under ambient atmosphere without degassing. [b]Monomer conversion was determined by ^1H NMR spectroscopy. [c]$M_{n,app}$ and $Đ$ were determined by SEC (THF as eluent) calibrated to polystyrene standards.

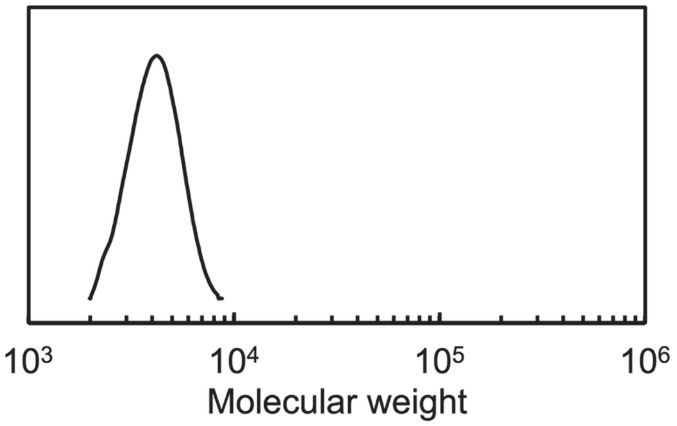

Figure S5. SEC trace of A6MPB after polymerization using TPMA as ligand.

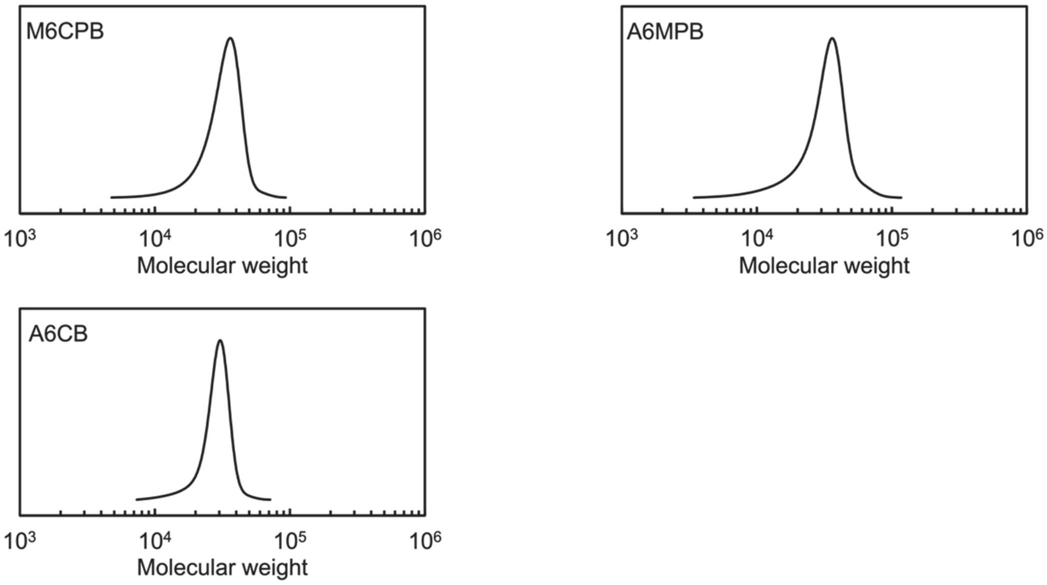

Figure S6. SEC traces of LCPs synthesized using different monomers (Table 5).

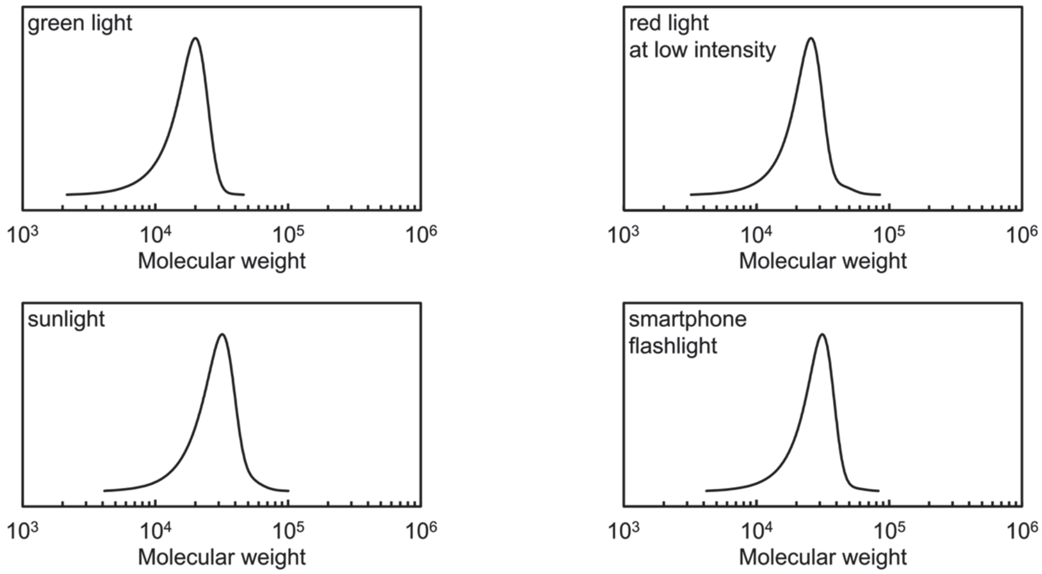

Figure S7. SEC traces of PM4MPB synthesized under green light (50 mW cm^{-2}), red light at low intensity (10 mW cm^{-2}), sunlight (approximately 1 mW cm^{-2} measured at 545 nm), and smartphone flashlight (40 mW cm^{-2} measured at 545 nm).

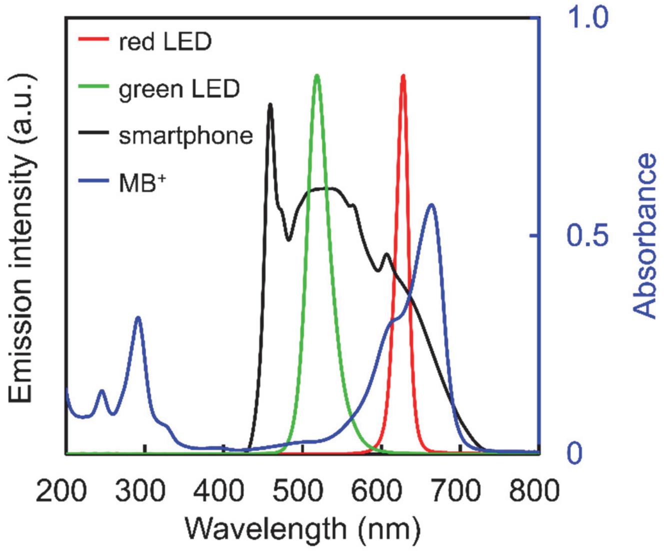

Figure S8. Emission spectra of the red LED, green LED, and smartphone LED flashlight used as light sources for photo-ATRP (left axis), together with the UV−vis absorption spectrum of MB^+ at a concentration of 1.0×10^{-5} mol L^{-1} in H_2O.
